# Infravec2 guidelines for the design and operation of containment level 2 and 3 insectaries in Europe

**DOI:** 10.1080/20477724.2022.2108639

**Published:** 2022-08-22

**Authors:** Emilie Pondeville, Anna-Bella Failloux, Frederic Simard, Petr Volf, Andrea Crisanti, Roya Elaine Haghighat-Khah, Núria Busquets, Francesc Xavier Abad, Anthony J Wilson, Romeo Bellini, Sarah Marsh Arnaud, Alain Kohl, Eva Veronesi

**Affiliations:** a MRC-University of Glasgow Centre for Virus Research, Glasgow, UK; b Institut Pasteur, Unit of Arboviruses and Insect Vectors, Paris, France; c MIVEGEC, Univ. Montpellier, IRD, CNRS, Montpellier, France; d Department of Parasitology, Faculty of Science, Charles University, Prague, Czech Republic; e Department of Life Sciences, Imperial College London, London, UK; f IRTA, Centre de Recerca en Sanitat Animal (CReSA, IRTA-UAB), Campus de la UAB, Cerdanyola del Vallès, Spain; g The Pirbright Institute, Woking, Surrey, UK; h Centro Agricoltura Ambiente “G.Nicoli”, Crevalcore, Italy; i Institut Pasteur, Genetics and Genomics of Insect Vectors, Paris, France; j UZH, Institute of Parasitology, University of Zürich, Zürich, Switzerland

**Keywords:** Vector biology, vector–pathogen interactions, guidelines, research infrastructures, insectaries, containment levels

## Abstract

With the current expansion of vector-based research and an increasing number of facilities rearing arthropod vectors and infecting them with pathogens, common measures for containment of arthropods as well as manipulation of pathogens are becoming essential for the design and running of such research facilities to ensure safe work and reproducibility, without compromising experimental feasibility. These guidelines and comments were written by experts of the Infravec2 consortium, a Horizon 2020-funded consortium integrating the most sophisticated European infrastructures for research on arthropod vectors of human and animal diseases. They reflect current good practice across European laboratories with experience of safely handling different mosquito species and the pathogens they transmit. As such, they provide experience-based advice to assess and manage the risks to work safely with mosquitoes and the pathogens they transmit. This document can also form the basis for research with other arthropods, for example, midges, ticks or sandflies, with some modification to reflect specific requirements.

## Introduction

The ongoing threats posed by arboviruses in many parts of the world, highlighted by the recent Zika virus (ZIKV, *Flaviviridae*) outbreak, have increased the interest in working with arthropod vectors of these pathogens. This has led to new insectary structures, or plans to build such structures, and different work practices that can make it difficult to interpret results or reproduce data. For example, the vast number of publications assessing mosquito vector competence to ZIKV revealed that the lack of method standardization largely hampers meta-analyses and therefore our understanding of vector competence [1]. It is therefore desirable to identify and standardize infrastructure design as well as procedures, either to adhere to common protocols or to be able to pinpoint differences.

Containment of arthropods (for example, those which are non-native to an area, those which are genetically modified (GM) and those which are infected with notifiable pathogens) as well as safe manipulation of pathogens are essential prerequisites for safe work and handling in this area of research. Historically, reported laboratory escapes of insect vectors leading to sustainable settlement and/or disease spread are extremely rare [2], and so far, there are no reports of laboratory escapes of insect-borne pathogens resulting in transmission outside the laboratory to the best of our knowledge. With the current expansion of vector-based research, and an increasing number of facilities rearing and infecting insect vectors for the study of vector–pathogen interactions, common measures for safe work are timely.

As part of Infravec2, a Horizon 2020-supported consortium integrating several European infrastructures for research on arthropod vectors of human and animal diseases, discussions between scientists involved in mosquito (and other arthropod vector) studies revealed the difficulties at various levels with regard to design and running of mosquito research infrastructures at containment levels (CLs) 2 and 3, as well as associated health and safety regulations. Some guidelines to work with arthropods have been previously published. While they constitute a useful resource, they were intended to give recommendations for structuring a public health entomology laboratory to guide prevention and control activities [3] or focused on arthropod work at CL2 [4]. Other guidelines providing advice on containment and controls for safe working with arthropods were intended to prevent escape and establishment and were not detailed with regard to infrastructures. In addition, they were written according to country (UK or USA)-specific regulations [2,5,6], which do not necessarily apply elsewhere in Europe.

The absence of detailed European guidelines to adequately set up and safely run a contained laboratory for vector research without compromising experimental feasibility can represent an obstacle for institutions establishing such new infrastructures in Europe. In addition, this results in the lack of harmonization/standardization across laboratories for operating procedures – which can impact the reproducibility of results. This was revealed by an Infravec2 survey carried out with scientists working on arthropod infection in contained facilities in Europe (corresponding to 21 laboratories), to assess community thoughts on the topic of ‘Containment procedures for working with infected arthropods and associated risks’. 70% of the participants perform arthropod infections on the bench or in climatic chambers, while others perform these experiments in MSC or HEPA-filtered isolators; 30% reported arthropod escapes in contained areas, and only 40% routinely use glove boxes (sealed containers) for sorting infected arthropods (Supplementary file 1). We believe that this survey demonstrated the urgent need for procedure harmonization, and how capacity building and networking can positively contribute to improving standards and work practices.

In this context, the Infravec2 project aimed to establish guidelines for the design and operation of insectary facilities, as well as for rearing, infection and management of infected mosquitoes (genetically modified or not) at CL2 and CL3. These guidelines, written by Infravec2 consortium expert members, reflect current good practice across European laboratories with experience in safe handling of different arthropod vector species and pathogens (Table 1). It provides experience-based advice to assess and manage i) the risks to individuals working with vectors and pathogens, ii) the risk of escape of arthropods, including infected arthropod specimens, into the environment, iii) the risk of establishment of exotic arthropod populations, particularly those capable of transmitting disease, in new regions, and iv) the risk of cross-contamination during experimental studies of arthropod infection. These guidelines should provide information to those interested in developing new insectaries as well as to those currently running such facilities. Beyond that, they constitute a first step toward unified procedures and logical workflows when setting up insectary structures. While this document focuses on mosquitoes and midges, it can also form the basis for studies on other arthropods, for example, ticks or sandflies.

**Table 1.** **Organisms and pathogens used by Infravec2 consortium members who contributed to these guidelines**. This guidance document is informed by the experience of the contributors and as such particularly reflects requirements for work with insects (mainly mosquitoes), which can be endemic, imported or exotic species. Moreover, these species can be genetically modified (*e.g*. lines containing a docking site, mutant for a gene, overexpressing anti-pathogen effectors, bearing gene drive systems, etc.) and/or be transinfected with endosymbionts, *e.g. Wolbachia.*

Organisms	Genera	Pathogens
Mosquitoes	*Anopheles*	Parasites *Plasmodium* ssp. (*P. falciparum, P. berghei, P. yoelii*)
	*Aedes*	Arboviruses (*Togaviridae/Flaviviridae/Bunyavirales*)
	*Culex*	Arboviruses (*Togaviridae/Flaviviridae/Bunyavirales*)
Midges	*Culicoides*	Arboviruses (*Reoviridae*)
Sand flies	*Phlebotomus*	Parasites *Leishmania* ssp., Arboviruses (*Phenuiviridae*)
	*Lutzomyia*	Parasites *Leishmania* ssp.

## Hazard groups and containment levels

Safety and risk assessment are central to insectary design. This involves risks associated with vectors as well as pathogens, where infections are part of experiments. Biological agents are categorized into hazard groups (HGs), also called risk groups (RGs), to reflect the risks they represent to laboratory workers and the environment. In the EU, guidelines on the HG/RG classification of microorganisms are given in Article 2 of the European Parliament and the Council Directive 2000/54/EC [7], and containment levels are defined in Annex V of the same directive. The CL required for work with GM organisms is similarly defined within the EU by Article 4(3) of Council Directive 2009/41/EC [8]. These definitions are typically further refined by national bodies such as the Advisory Committee for Dangerous Pathogens (ACDP) in the UK, the Comisión Nacional de Bioseguridad (CNB) in Spain, the Agence Nationale de Sécurité du Médicament et des Produits de Santé (ANSM) in France, the Zentrale Kommission für die Biologische Sicherheit (ZKBS) in Germany, the Bundesamt für Gesundheit (BAG) in Switzerland among others. Similar classification systems may be used to classify risks to animal health, as defined by the UK’s Specified Animal Pathogens Order, for example [9]. Hazard Groups range from HG1 to HG4 and determine, in general terms, the CL required for the safe handling and manipulation of microorganisms (from CL1 to CL4). CLs reflect a set of containment principles and precautions, as well as actions and procedures to work with pathogens in an enclosed laboratory. They are also named biosafety levels (BSL-1 to BSL-4) or pathogen or protection level (P1 to P4) depending on location/country, which needs to be verified locally.

CL and procedures required for safe laboratory work with biological agents or GM organisms generally reflect not only the intrinsic risk of the agent (as indicated by its HG rating) but also the nature of the work, and additional more stringent measures are frequently required for studies on infected arthropods [10]. The consequences of genetic modification of biological agents, or of arthropods, also need to be taken into account when assessing risk and legal requirements. As such, HG, CL and GM regulations need to be carefully evaluated when designing insectaries and planning work within. The provision of a safe working environment and the assessment and management of risk resulting from work is typically a legal obligation for employers. The guidelines provided here are meant to support decision makers in the process of ensuring that their workplace offers adequate protection to workers, the general public and the wider environment when working with biological agents within arthropods.

A general overview of the four different HGs and CLs is provided in Table 2. In practice, given the nature of pathogens and arthropods, most work involving infected mosquitoes will occur at CL2 or CL3. Risk assessments must consider the arthropod species, its biology, and ecology, the pathogens it can transmit as well as epidemiological data. For example, work with autochthonous species that could harbor/transmit a given pathogen or with highly invasive exotic species may require studies to be carried out at higher biosafety level.

**Table 2.** Summary of hazard groups (HG) and containment levels (CL) for the purpose of this document.

HG	Risk to individual	Risk to community	Minimum CL	Laboratory practice	Safety equipment
HG1	None or very low	None or very low	CL1	Standard microbiological procedures	- Bench work - Personal protective equipment (PPE): lab coat and gloves, eye and face protection if needed
HG 2	Moderate	Low	CL2	CL1 plus: - Limited access - Biohazard signs - Sharps bins Precautions - Hand washing sink near to exit - Risk assess waste and/or medical surveillance	CL1 plus: - Microbiological safety cabinets (MSC) when potential aerosols/splashes - Autoclave available in the building
HG 3	High	Low	CL3	CL2 plus: - Controlled access - De contamination of waste - Clothing change before entry and protective clothing	CL2 plus: - PPE: Full body suit, glasses - MSC/HEPA-filtered isolators for all work - Alternatively, respiratory protection as needed (full face masks, masks/protective glasses, powered air purifying respirators). - Physical separation of the laboratory - Double-door access and selfclosing, airlock entry - Negative air flow - No recirculation of air - Autoclave available in the laboratory - HEPA Filtration at all exhaust air. - Pressure monitoring - Safety systems for lone working; monitoring/CCTV for laboratory spaces. - Optional: Shower facility (or air shower).
HG 4	Very high	High to very high	CL4	CL3 plus: - Identifiable clothing change before entry - Chemical shower on exit - All material decontaminated before exit	CL3 plus: - Class III MSC or class I/II with air-supplied positive pressure suits - Double-ended autoclave (through wall), effluent system - HEPA-filtered air at the entry and the exhaust. - Separate or isolated zone - Monitoring/CCTV and safety systems for all aspects of work; safe storage of pathogens and biological material.

The containment requirements for specific biological agents will depend on their HG classification in the country of use. The same applies to requirements for arthropod containment and the classification of work involving infected arthropods. As such, locally applicable rules such as licensing, validation procedures, risk assessments (RAs), code of practice (COP) and standard operating procedures (SOPs), and specific structural requirements for buildings where work is to be conducted in containment need to be devised, agreed upon, and implemented.

The following areas need to be considered in assessing the overall risk of an activity involving infected or GM mosquitoes, but also other arthropods:

- The risk of, and posed by, mosquito escapees to the environment, especially if potentially leading to the establishment of a population of genetically modified and/or non-native vectors. This should include not only the likelihood of escape but also the risk of establishment given the local environmental conditions and their impact on the potential for the survival and reproduction of the species in question.
- The risk posed by the mosquito to laboratory workers: for instance, the potential for transmission of biological agents inadvertently present in mosquitoes collected at field sites where other pathogens are circulating.
- The public health, animal health or environmental risk posed by the biological agents that mosquitoes are infected with, to laboratory workers and the environment.
- The risk posed by the pathogen to arthropod colonies (for example, contamination of non-infected strains or affecting fitness of the experimentally infected arthropod colonies).
- The risk of cross-contamination when performing infection experiments with different vectors and/or pathogens.
- The risk of pathogen transmission between persons, also from a facility staff/visitor to community.
- The availability of treatments and preventive measures.

When working with mosquitoes and pathogens that are genetically modified, the CL required may need to be reevaluated depending on the planned activities and on the nature of the transgenic modifications, such as driving transgenes that are more likely to persist [2,11].

In the context of these guidelines, we focus on CL2 and CL3 insectaries. Specifics for each CL are as described below.

## The CL2 Insectary

### General requirements

- Adequate information, instructions, training and supervision of all workers and visitors.
- Access restricted to users (PIN keypad, access badge or physical key, biometric reader).
- Access to autoclave in building plus validated inactivation and waste disposal procedures.
- Protocols for disinfection in place; waste streams according to national regulations.
- Vector control measures such as traps to prevent the intrusion/escape of arthropods are recommended throughout insectary rooms (light traps, attractor tape, fly catcher [electrical or mechanical]). Traps can be checked regularly, and numbers of vectors recorded.
- Display (in the laboratory) of key standard operating procedures (SOPs) and emergency procedures.
- Equipment for the safe storage of biological material (e.g. locking freezers).
- Display of safety signs on laboratory door including authorized persons and biohazard signs (*e.g*. genetically modified organisms).
- Record keeping for RAs, SOPs, and other relevant records.
- Monitoring of activities to ensure implementation and effectivity of RAs, controls, and SOPs.

### Layout

Any potential arthropod escape routes from the insectary need to be controlled, for example, curtains to stop escapes from reaching outside/anterooms or mesh for drainage/air vent systems. Barriers such as mesh need to be of an appropriate size to contain the relevant arthropod. For example, adult *Culicoides* biting midges or sand flies may escape through mesh hole sizes that are effective for containing adult mosquitoes. The same principle applies when considering mesh sizes needed for blocking drains to prevent the accidental release of eggs.

#### Entry of insectary rooms

**Figure 1.**
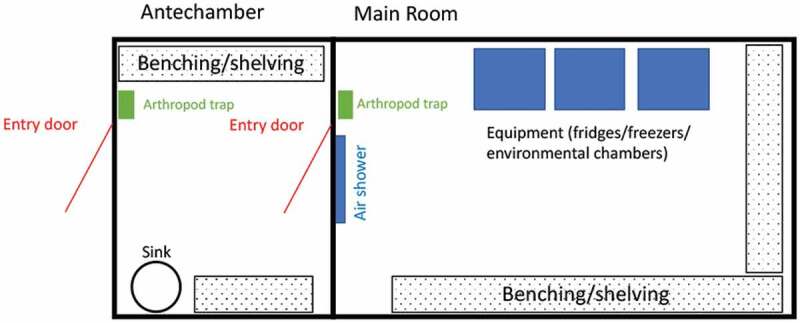
**Layout for a CL2 insectary**. The main room is either humidity and temperature controlled (in this case, electric equipment must be placed outside the humidity room, in the antechamber or in a room close by) or equipped with environmental chambers. Air showers/curtains can be useful upon exit to avoid escapes. Benching and shelving can be stacked up to gain storage space.

Insectaries should generally consist of an antechamber or preparation room for activities such as material preparation and storage which should also contain a sink for hand washing and water supply, as well as rearing rooms for housing and working with arthropods (Figure 1). A freezer for killing adult mosquitoes within their primary containers is strongly recommended. To limit escapes in the antechamber, a curtain can be placed before the door inside the main room. A lobby with double doors before the antechamber may add an extra layer of containment, if required. Cooling the lobby (to 4°C) may be effective at limiting the movement of certain species, particularly tropical species. Alternatively, an air shower can be placed inside the main room above the exit door.

#### Secondary containment

Two design approaches are recommended to contain and rear arthropods: a temperature/humidity-controlled room or environmental chambers.

**Figure 2.**
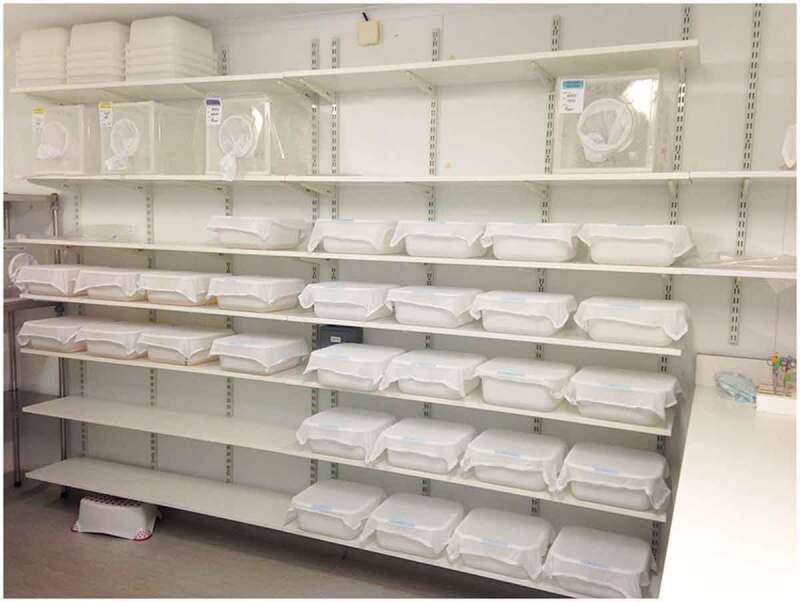
Stacked shelves housing breeding pans and cages in a humidity- and temperature-controlled room.

Option 1: the temperature/humidity-controlled room. This approach, sometimes called controlled-temperature rooms (CT rooms), is preferable if large numbers of arthropods are handled, as it uses space more efficiently (Figure 2) but there is a high risk of fungal or bacterial contamination if rooms are operated at high humidity. This risk may be reduced by regular cleaning of all surfaces, using appropriate benching material, wall coatings or materials and specialist paint, although such choices will be associated with a cost. Surfaces such as benches need to be impervious to water and resistant to alcohol or solvents/cleaning products to allow thorough decontamination. Care should be taken that the chemicals used do not affect vector survival or behavior, for example, through chemical traces left in breeding pans or released into the room. This solution requires dedicated engineering oversight and maintenance. Lighting is crucial and windows, if any, should be covered to ensure a controlled environment. Light timers are useful to provide regular day/night cycles. It is critical that in such an environment, any equipment that is sensitive to heat or humidity is adequately protected; if this is not feasible it should be stored separately when not in use.

**Figure 3.**
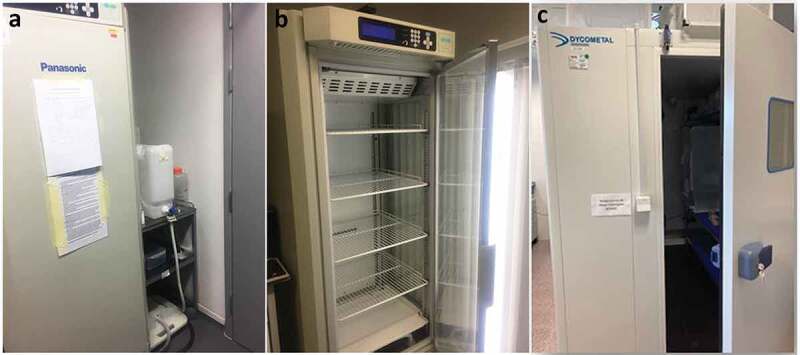
**Examples of environmental/climatic chamber with humidity, temperature, and light control**. (a, b) Climatic chamber connected to a water container for water supply and to a waste container to collect wastewater. (c) Walk in climatic chamber.

Option 2: environmental chambers. Environmental chambers (Figure 3a,b) provide additional containment and generally allow environmental conditions (temperature, humidity, and light) to be controlled more reliably and cheaply than an entire controlled-temperature room. However, they represent a relatively inefficient use of space and frequently limit the volume of work that can be carried out at any given time. Alternatively, walk in climatic chambers (Figure 3c) can be used to handle a higher number of mosquitoes and perform some specific assays that require space and controlled environmental conditions (*e.g*. behavior assays). Chambers also require regular maintenance by users. This normally includes regular cleaning, and for controlled-humidity environmental chambers may also include topping up with deionized water and removing wastewater, although for an additional cost chambers can be directly connected to a water supply and draining system to decrease the maintenance. A secondary internal glass door makes it easier to identify any breaches of primary containment without breaching secondary containment. Environmental chambers provide a very good solution to host infected mosquitoes.

For insectaries housing mosquitoes that carry a driving transgene, an extra containment from other mosquito colonies constitutes a contingency to reduce the risk of contamination of other mosquito colonies of the same species.

#### Manipulation room

**Figure 4.**
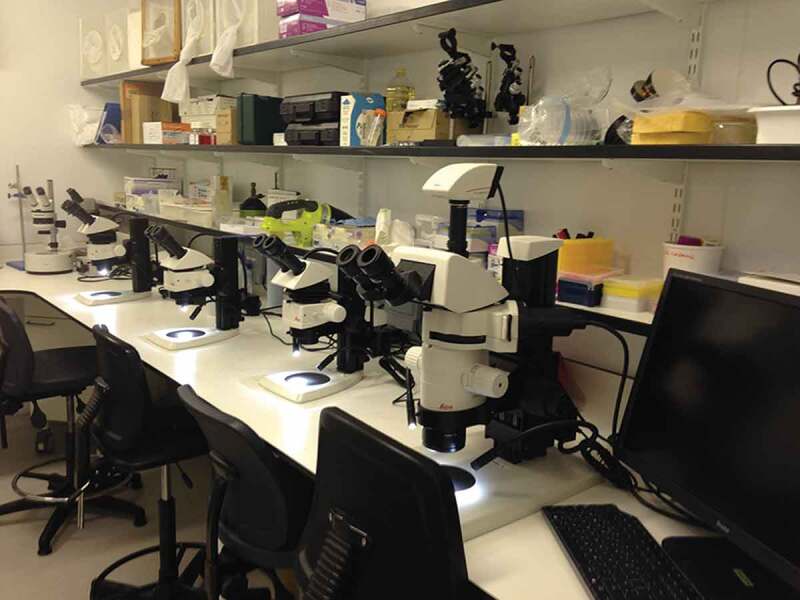
Room with stereomicroscopes and material for mosquito manipulation, such as dissection and injection.

A separate room is normally used for manipulation, dissection and injection of mosquitoes, as well as for any other procedures requiring stereomicroscopes and other non-portable equipment (such as freezers to kill arthropods, refrigerators or CO_2_ supplies for anesthesia, and freezers for storage of material). An example of such a room is shown in Figure 4.

### Containment and manipulation of non-infected mosquitoes or other arthropods

**Figure 5.**
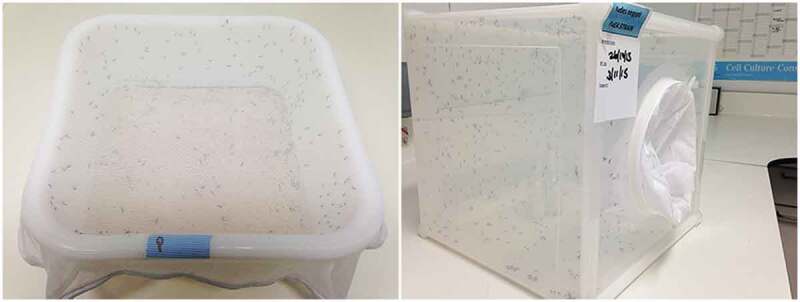
**Primary containers for non-infected mosquitoes**. Left panel, breeding pan covered with a weighted net for mosquito larvae. Adults can emerge in pans and are further aspirated. Right panel, typical cage for adult mosquitoes with plastic and meshed panels plus a mesh sleeve allowing access.

The container directly holding the arthropods is defined as the primary containment. For mosquito larvae, this is normally a rearing pan covered by a weighted net (Figure 5) or rigid transparent cover. For the latter, pans can be stacked up to save space. Mosquito pupae must be picked up daily and transferred into small cups and placed inside a cage to allow the emergence of adults into adult primary containment. If pans are covered by a net, adult mosquitoes can be alternatively aspirated after emergence and transferred into adult primary containment. This solution is time saving, compared to pupae transfer. Containers such as mesh cages can be used for large numbers of adult mosquitoes; smaller cardboard containers with mesh on top for smaller numbers of adult mosquitoes. Other arthropods may require other procedures. Relevant information should be written on primary containers (species and other relevant details, for example, origin, treatment, etc.). For adult arthropod manipulation, insects can be anesthetized within their primary container in a fridge (4–6°C) for 10–20 minutes and then transferred to a glass Petri dish placed on an ice bucket. Alternatively, they can be aspirated from a cage with a small electric hand-held aspirator with a mesh cover on the inlet and transferred into a plastic container (Falcon tube for instance) placed in ice to knock down mosquitoes. Note that a mouth pooter should not be used to avoid user allergy to mosquito scales in the long term as well as risks with infectious agents. Where mouth pooters are needed due to the delicate nature of insects, it has to be equipped with adequate filters (*e.g*. HEPA filters). Chill tables/ice packs or CO2 anesthesia tables are good alternatives for working with large numbers of mosquitoes. Note that CO2 may be the only effective option to anesthetize some mosquito species originating from cold and temperate regions, as these may be tolerant to low temperatures and may remain active. Incubation times and methods described above have to be assessed and adapted for individual mosquito species/populations, as well as for other arthropods, and options will depend on species’ biology and available facilities. For the transport of live mosquitoes (GM or not) outside of the CL2 facility, all mosquitoes must be transported in double-containment, and should include information on the strain and emergency contact details.

### Containment and manipulation of HG2-infected arthropods or for quarantine measures

Pathogen-specific risks must be considered, including potential pathogens and the risk of transmission for a given procedure. This requires careful consideration of relevant biosafety measures and biosafety legislation for handling of pathogens, for example, whether infectious blood feeding on the bench is acceptable or whether additional measures are necessary. For instance, to prevent any possible spillage of infectious blood on the bench or surface, due to the rupture of the feeding membrane, it is suggested to hold feeding cages on a secondary lid-free container.

**Figure 6.**
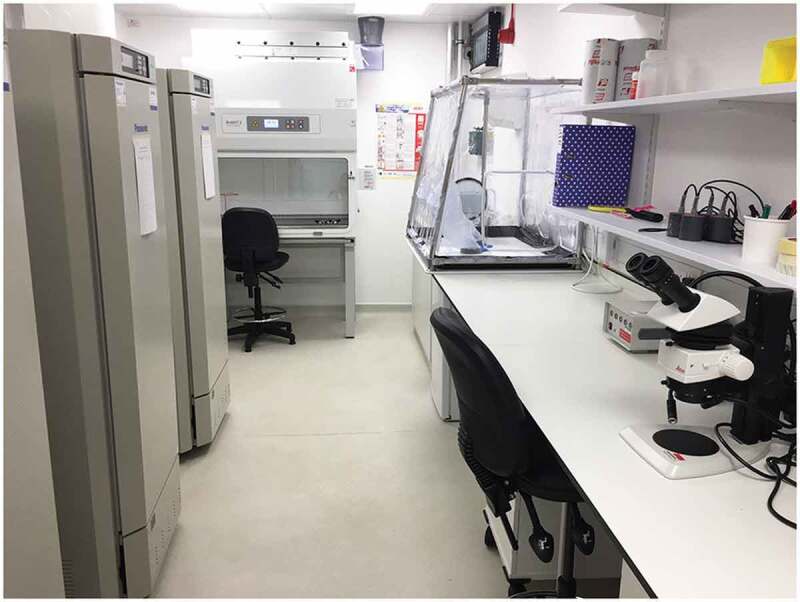
**Example of CL2 insectaries dedicated to mosquito infection with HG2 arboviruses**. The room is equipped with a class II MSC, to prepare the infectious blood and blood capsules, a glove box, which is a sealed container allowing user to manipulate flying arthropods inside the box without breaking containment, a bench with stereomicroscopes for mosquito dissection, environmental chambers, a freezer to freeze all solid waste before taking out for autoclaving, a sink to dispose of chemically inactivated waste, a UV light trap, a fridge and storage shelves/furnitures.

Ideally, mosquitoes infected with HG2 pathogens or mosquitoes in quarantine should not be reared in the same room as non-infected mosquitoes. If this is not possible, all procedures should be as those for infected mosquitoes, including when dealing with escapees. A class II microbiological safety cabinet (MSC) in the infection room is useful to prepare infectious blood meals and avoid movement of infectious material from one room to another. An example of CL2 insectaries dedicated to HG2 arbovirus infection is shown in Figure 6.

**Figure 7.**
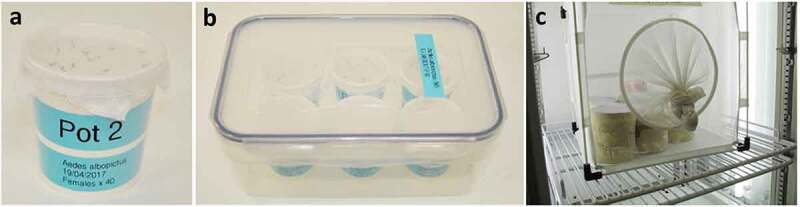
**Example of primary and secondary containers**. (a) Small and secured primary container with a netted lid. (b) Primary containers in secondary container for transport. (c) Primary containers in meshed cage inside environmental chamber.

Generally, uninfected mosquitoes are reared outside of the CL2 infection insectary and adult mosquitoes are transferred in small and secured primary containers within a secondary container (Figure 7(a, b)) before infection. It is important to establish that procedures used for infected mosquitoes are adequate for minimizing and dealing with escapes. For the primary containment of mosquitoes, we recommend secured pots, strong enough to withstand, for example, wet sugar cotton or blood feeding capsules, which can be easily transferred. Importantly, the number of arthropods per container should be relatively small and fixed (*e.g*. 20 to 50 individuals per box) and recounted to confirm that no escapes have occurred during their manipulation. When aspirating mosquitoes from a cage to transfer them to smaller containers, we recommend anesthetising mosquitoes on ice/CO_2_ before placing them in the container to make sure the number transferred. This might not be feasible if the risk of compromising the mosquitoes’ fitness has any implications, for example, mosquitoes that have been micro-injected with transgenes may already have impaired fitness and need to be handled with greater care until the new GM colony is established. Mosquito boxes must be marked with relevant information such as the number/species of mosquitoes, experimentator name, pathogens, date, etc. The climatic chamber provides secondary containment and should have a see-through door or internal glass door to reduce the risk of accidental escape before opening (Figure 3a, b). If a humidity- and temperature-controlled room is used or a chamber without a glass door, mosquito primary containers can be placed into meshed cages providing secondary containment (Figure 7c).

For adult mosquito manipulation, insects can be anesthetized in their primary container in a fridge for 10–20 minutes and then transferred on a Petri dish placed on an ice bucket. Aspiration of flying and infected mosquitoes should be avoided to decrease escape risk. However, for infection with some pathogens such as *Plasmodium falciparum*, recently engorged mosquitoes should not be cold-anesthetized to select blood-fed females as infections could be impacted. Here, mosquitoes are fed in a cage and non-blood-fed mosquitoes are removed after the blood meal using a small electric aspirator [12]. Note that CO2 may be the only effective option to anesthetize some mosquito species originating from cold and temperate regions. Gloveboxes, which are sealed containers allowing users to place their hands into the gloves and perform tasks inside the box without breaking containment (Figure 6), are generally a good preventive measure to contain and avoid escapes of infected or presumably infected mosquitoes or other flying arthropods during procedures that require manipulation outside of primary containment. When using a glove box, the primary container should be placed on an ice bucket to move it between the fridge and the glove box to avoid mosquitoes waking up during the process.

Once anesthetized and placed on ice, mosquitoes can be manipulated on the bench and should be manipulated one by one to minimize the risk of escape. When using forceps or sharps such as the capillary needles used for microinjection systems, extra caution should be taken to avoid injuries (do not cross hands, do not recap needles, instrument placement, etc.). Breeding of infected mosquitoes might be required for specific experiments, for example, studies on vertical transmission or the impact of infection on arthropod fitness. In this case, larvae can be reared in small containers tightly covered by secured mesh and placed in larger boxes also covered by a secured net. For transport, if possible, close the larger box with a lid to avoid the risk of spillage of the water in the rearing container. Pupae should then be transferred into a small cup or flask inside primary adult containment for emergence. Gloveboxes as described above or larger meshed containment to house pans and cages can provide a layer of security for manipulation during rearing (*e.g*. opening primary container to place/remove egg laying paper).

### Feeding devices

**Figure 8.**
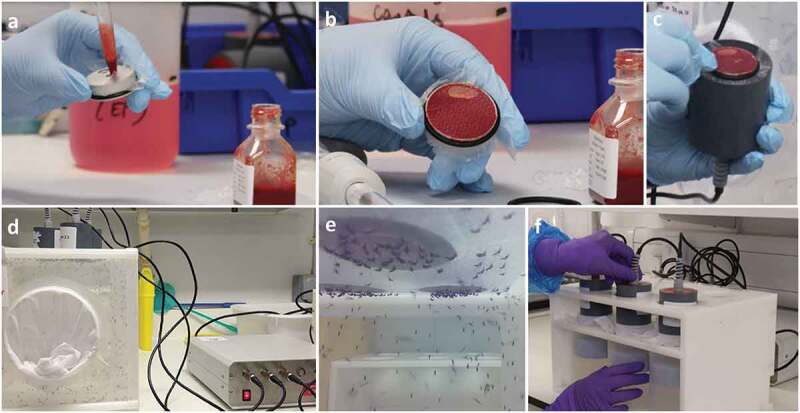
**Mosquito blood feeding with non-infectious or infectious blood meals**. (a) The blood is poured into capsules. When blood is to be spiked with pathogen, this step should be performed in an MSC. (b) Feeding capsule filled with blood and covered by an artificial membrane. (c) Blood capsule connected to the heating unit. (d, e) non-infectious blood feeding of mosquitoes in cage. (f) Infectious blood feeding of mosquitoes in small and secured primary containers.

For routine feeding of male and female mosquitoes, cotton imbibed with 10% sugar solution can be placed inside cages or on top of smaller containers. When mosquitoes are infected with pathogens that can be transmitted through mosquito saliva, sugar cotton must be considered as infected and disposed accordingly. Blood feeding in CL2 is frequently carried out on bench spaces. However, an accidental spillage of infectious blood during the blood feeding must be treated according to safety procedures and waste treated as infectious waste. Laboratories frequently use temperature-controlled devices (such as Figure 8) to feed mosquitoes with blood heated at 36–37°C. The blood (infectious or not) is poured into capsules, with one side covered by an artificial feeding membrane at the bottom and the capsule then connected to the temperature control heating device unit (Figure 8(a–c)). Artificial feeding membranes or parafilms can be used, although animal skin or membranes (*e.g*. pork intestine and chick skin) may increase feeding rate – and sometimes be the only option – for some species or recently field-isolated colonies. The use of human blood requires a specific risk assessment and user vaccination (*e.g*. Hepatitis B) might be required if the blood is not previously screened for blood-borne pathogens. If the blood meal is not infectious, mosquitoes are usually fed in cages (Figure 8d,e); and if it is infectious, mosquitoes are fed in smaller and secured primary containers with a netted lid (Figure 8f) to facilitate anesthesia in a fridge and further selection of engorged females on ice. Mosquitoes can also be directly fed on animals, for instance, for transmission studies. In this case, prior ethical approval is needed according to each country or regional policies (note that these guidelines do not cover vertebrate animal work).

**Figure 9.**
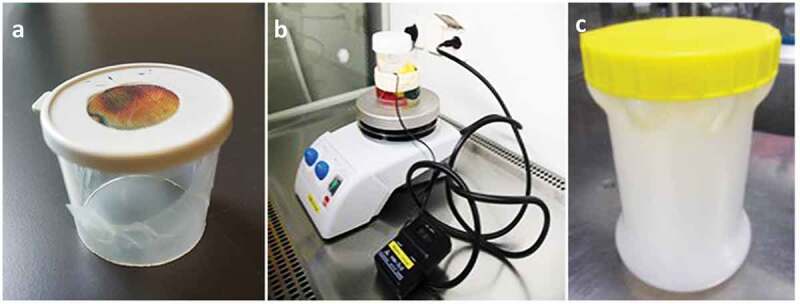
**Enclosed feeding device system for *Culicoides***. (a) Primary container ‘netted feeding chamber’ where *Culicoides* will be kept during blood meal. (b) Secondary container, ‘infectious blood reservoir chamber’ in a water bath and magnetic stirrer with temperature probe. (c) Transport box for movement of infectious items between rooms.

Other feeding methods may be more appropriate for other arthropods. For example, for *Culicoides* midges, one method is to aspirate live *Culicoides* with an aspirator and transfer them inside a plastic ‘feeding chamber’ (Figure 9a) which has a netted lid and a membrane (as described above) on the bottom side. The feeding chamber then needs to be transported to the CL2 infection room for oral feeding with pathogen-spiked blood. This primary container will be inserted into a secondary container, the ‘infectious blood reservoir chamber’ containing infectious blood and a magnet to allow constant mixing of pathogen and blood. These two containers are placed onto a large petri dish containing water pre-warmed to 25–30°C. All three containers are allocated on a heating magnetic stirrer pre-warmed to 25–30°C. The temperature is checked by a probe inserted into the water bath during feeding (Figure 9b).

### Pathogen inactivation and disposal of insectary waste

Waste inactivation and disposal is generally regulated by national and/or local biosafety regulations. Solid insectary waste may fall in Animal By-Products (ABP) waste. In the EU as well as in the UK, ABP (entire animal bodies, parts of animals, products of animal origin or other products obtained from animals that are not fit or intended for human consumption) must be segregated and identified for disposal via appropriate routes, generally incineration as ABP. In the EU, ABP and associated regulations are defined in Regulation (EC) 1069/2009 and (EU) 142/2011 [13]. In the UK, these regulations are administered and enforced by the Animal By-Products (Enforcement) Regulations 2011. Arthropod killing and pathogen inactivation procedures, as well as disinfectant agents, must be validated if not already established. It is recommended that all solid waste from mosquitoes, even when not infectious, should be discarded into autoclave bags, left overnight in a freezer (to kill all mosquitoes and avoid potential escapes before autoclaving), autoclaved and discarded. This includes all materials used, *e.g*. gloves, mosquito debris, egg laying papers, wet cotton, cotton rolls, larval food waste, etc. Water from larval rearing should be filtered (for example, through 50 μm Nitex cloth mounted on a hoop fishing net) to remove any embryos/larvae/adult escapees before it enters the wastewater system. The larval containers can also be scalded with or immersed in hot water to kill any eggs/larvae, or materials can be frozen at −20°C overnight. Filter mesh sizes, freezing times and the effectiveness of hot water/immersion time will depend on arthropod species.

For HG2-infected solid waste to be transported to an autoclave outside the insectary, additional controls such as inactivation by spraying with 70% ethanol may be considered before freezing. Solid and liquid waste (including ice) can also be inactivated by overnight immersion in disinfectant (*e.g*. Virkon), subsequently sieved (liquid to drain) before transferred into autoclave bags for autoclaving (solids). All disposable protective clothing is placed in autoclavable bags and autoclaved. Sharps should be discarded in sharps bins filled with a disinfectant and sent directly for incineration.

If using metallic blood capsules, some disinfectants can be corrosive and damage capsules during longer period of soaking (*e.g*. more than 10 min in Virkon). In this case, capsules filled with infected blood can be sprayed with the disinfectant, then immersed in water in a lidded container for transport to autoclave, autoclaved and washed for reuse. Dissection tools should be surface disinfected before drying. Reusable materials that cannot be autoclaved, such as primary plastic containers and sample containers (*e.g*. tubes or tube boxes), can be immerged overnight or sprayed copiously with an inactivating agent before being removed from the CL2 infection insectary. Non-inactivated samples (*e.g*. mosquito tissues) can be transported in double containment to a CL2 laboratory for further processing and according to specific and local regulations.

### Personal protective equipment (PPE)

PPE should be determined by risk assessment. For work with non-infected arthropods, regular laboratory protective clothing such as lab coat and gloves are required. In temperature- and humidity-controlled rooms, a disposable apron may be worn in place of lab coat. For work with pathogens and infected arthropods, laboratory protective clothing such as disposable lab coats, gloves and safety spectacles are required. For arthropod manipulation such as dissections, where there is a higher risk of worker exposure (*e.g*. projections) to infected material, FFP3 masks should be considered as well as wearing disposable apron and sleeves (on top of a disposable lab coat). Puncture-resistant gloves can also be worn when using sharps, such as dissection forceps, to reduce sharp-associated risks. Pathogen-specific risk assessments for at risk staff, *e.g*. pregnant and/or breastfeeding women, should be carried out.

### Emergency measures

A key concern in the insectary is the escape of arthropods (especially if infected). Individual escapes or low numbers can be dealt with by direct killing (handheld zappers or fly catchers are useful). Larger numbers are more difficult and may necessitate the use of formaldehyde-based fumigation or Vapor Hydrogen Peroxide (VHP) decontamination by fumigation though this would kill all arthropods in the insectary and thus negatively affect operations for long periods. As mosquitoes require frequent sugar feeding for hydration, larger escapes may be more effectively dealt with by removing any source of free water from the room, sealing the affected room and waiting for mosquitoes to die (typically within 1 week post escape) before reentering and cleaning the concerned room, but this needs to be assessed for each species. If possible, increasing room temperature and decreasing humidity can be effective. For example, if mosquitoes escape into an environmental chamber, the temperature can be set to 50°C for a minimum of 2 h to kill mosquitoes before opening the climatic chamber. To have confidence in the effectiveness of such methods, they must be validated using trial runs with non-infected arthropods before any infectious work begins. Spills with pathogen-containing material need to be considered and dealt with according to local regulations.

In order to facilitate the workflow for accessing CL2 laboratories, a checklist summarizing all the standard procedures required in relation to the topics analyzed in these guidelines is provided (Supplementary File 2, Table S1). This checklist can be shared with the staff working in this environment.

## The CL3 insectary

The CL3 insectary operates at a higher safety level than CL2 and requires additional safety measures that inform the layout and operation of such structures.

### General requirements

- Adequate information, instructions, training, and supervision of all staff, including direct and continuous supervision (timely training updates and evaluation) when required.
- User access restrictions required (pin and/or swipe card and/or biometric readers among others) and dedicated protective clothing/PPE.
- The laboratory must be protected with an intruder alarm, according to pathogens used and national regulations.
- Sealed laboratory (secondary containment or barrier) and HEPA filtration of air outflow to prevent any accidental release of pathogen.
- Negative pressure gradient environment (with pressure alarm system), to be adapted to sensitive species such as sand flies.
- Access to autoclave within the CL3 suite or double-ended; and if required access to incinerator or tissue digester; validated inactivation and waste disposal procedures in place.
- Protocols for disinfection in place; waste streams according to national regulations.
- Vector control measures such as traps to prevent intrusion/escape of arthropods are recommended throughout insectary rooms. These can include light traps, attractor tape, fly catchers (electric or mechanic), mesh (according to arthropod size; on vents, for example); if sinks/pipes are present liquid traps/barriers should be considered.
- Display or direct access to RAs, COP, SOPs, and emergency contacts in the laboratory, at all times.
- Safe storage of biological material must be ensured. Pathogens falling under HG3 regulations can only be used within such a facility and must be handled accordingly.
- Display of safety signs on laboratory door including authorized persons and biohazard signs.
- Record keeping for training, RAs, SOPs, and other processes.
- Record keeping of all strains/colonies/clones/variants handled in the facility.
- Monitoring of activities to ensure implementation and effectivity of risk assessments, controls, and procedures.
- Vision panels and/or web cams/CCTV to allow supervision of workers within the CL3 laboratory.
- Transport measures (containers, etc.) for movement of samples between rooms and in/out of the facility. The exit of samples from CL3 to CL2 should be carried out according to validated disinfection/inactivation procedures.

### Layout

#### Access to CL3 insectaries

**Figure 10.**
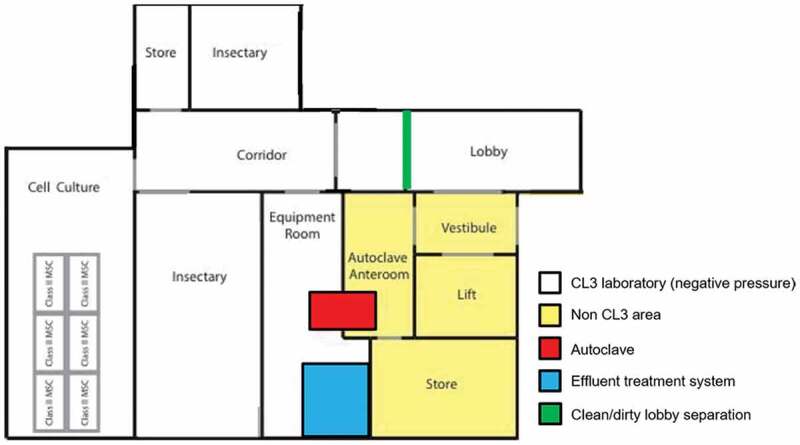
**Layout of a CL3 insectary**. Access to the CL3 area is via a lobby divided (by a door or simply by a mark on the floor) into a clean (entrance side) and a dirty (CL3 corridor side) area. After the lobby, a corridor can give access to insectary room(s) and a cell culture room which can be used to prepare and store pathogens. Mechanisms to avoid simultaneous opening of doors should be put in place. A double-ended autoclave allows autoclaving waste from the CL3 area and removal of autoclaved waste from outside the CL3 area. Store and equipment rooms are optional. Chemically inactivated liquid waste can be autoclaved or discarded using an effluent treatment system.

The CL3 facility must be separated from other activities. It is generally suggested that a CL3 insectary possesses a lobby to put on/off PPE and which also provides another level of segregation as shown in Figure 10. Access needs to be controlled and restricted; depending on design specific access to the insectary may require additional permission. The lobby should be separated (by a door or simply a mark on the floor) in two areas: clean (entrance side) and CL3 area (CL3 corridor side). The clean area can be used for personal belongings and clean PPE but also contains a handwashing sink. The CL3 area is used to store PPE (*e.g*. protection glasses and suit) in use and should contain an autoclave bin to discard any used PPE. Shoe covers or CL3-specific shoes should be worn when entering the CL3 area and removed before leaving to the clean area.

When entering and leaving a CL3 insectary, work must be logged (experimenter, pathogen, experimental details, time in and out). The entrance door pressure gauge reading must show a negative value before entry to indicate that the pressure gradient is adequate (red/green light warning systems or similar may be useful). Rules for pair/lone working such as alert devices apply to CL3 insectaries as much as standard CL3 laboratories. An interlocking system must be used to prevent simultaneous opening of the outer (lobby) and inner (corridor) doors. A cooled lobby at 4°C or an insect trap in the lobby can also be useful to limit the risk of escape, especially of tropical arthropod species.

#### Insectary rooms

After the lobby, a corridor gives entry to the insectary rooms (an air shower can be a useful addition, to remove any arthropods hiding on clothing). We recommend a layout with climatic chambers (Figure 3) as in addition to allowing variable conditions (temperature, humidity, and light) these are easier to maintain, can be replaced easily and provide additional containment. However, these require regular maintenance (cleaning, filling with water, and collecting wastewater). A waste draining system is a critical issue in this setting as liquid waste necessitates decontamination before removal from the CL3 insectary, either by chemical inactivation or by a specialized effluent treatment system. A fridge may be present in the insectary room to allow anesthesia of mosquitoes in their primary containment before sampling/manipulating them (see below), but this needs to be individually assessed depending on procedures and research activities. Depending on space, the room can accommodate climatic chambers and bench space for manipulation of mosquitoes such as blood feeding and dissection (in an HEPA-filtered isolator, for example, see below). If more than one room is available, activities can be split either by the type of experiment (blood feeding versus dissection) or by the pathogen used.

#### Other rooms and equipment

Besides insectary rooms, a cell culture room with incubators and class II MSCs is useful to grow and manipulate/prepare pathogens *(i.e*. parasites and viruses). A class II MSC will also be required to prepare infectious blood to infect mosquitoes. An equipment room can be used to place freezers (−80°C to store viruses, for example) but also autoclave (if double ended with exit and externally vented in an anteroom located out of the CL3 laboratory) as well as an effluent treatment system, if required. All equipment such as freezers, centrifuges, etc., should be planned into layout during the early design stages, importantly power consumption and heat generation may need to be taken into account. Given costs and size considerations, room usage should be maximized. However, only a minimal amount of material and consumables should be stored directly inside the CL3 laboratory to avoid overcrowding in order to facilitate detection of any potential escapees and disinfection.

### Containment and manipulation of infected mosquitoes

**Figure 11.**
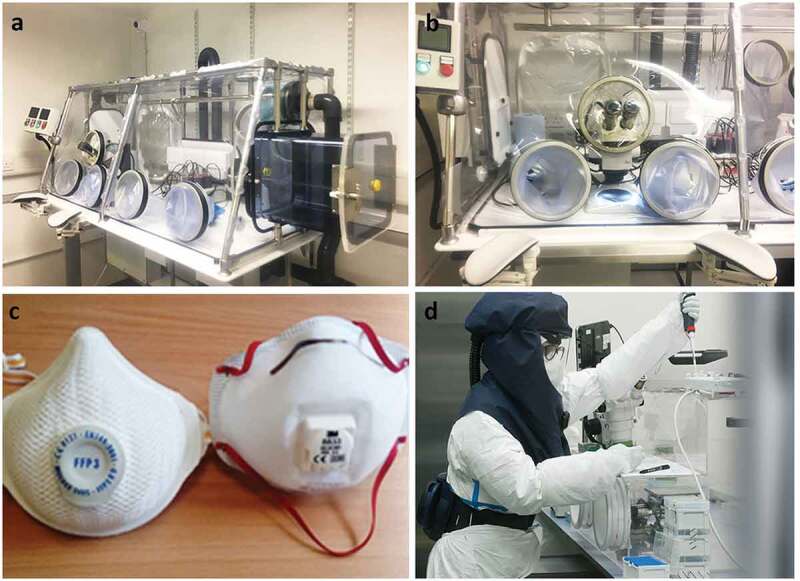
**Protective equipment to work with HG3 pathogens**. (a) HEPA-filtered isolator working under negative pressure for manipulation of HG3 infectious material which can be aerosol transmitted. A hatch allows material entry while work is ongoing. Height is adjustable. (b) Incorporated stereomicroscope to dissect infected mosquitoes in HEPA-filtered isolator. (c, d) Filtering face piece (FFP) 3 masks or Purifying Air Powered Respirators (PAPR) that can be used in CL3 in place of a HEPA-filtered isolator.

Transport, containment and feeding devices of mosquitoes are similar to HG2 activities. In addition, manipulation of mosquitoes infected with HG3 pathogens requires protection of users against aerosol transmission risk, where known. This can be achieved by performing all manipulations including blood feeding inside a HEPA-filtered isolator working under negative pressure (Figure 11a,b). These can also be constructed to include stereomicroscopes for dissection. These designs provide a good containment (for both mosquitoes and pathogens) but render fine manipulations (dissections and injections) difficult, which can be problematic when a large number of mosquitoes are required for an experiment. In addition, the negative pressure inside the isolator can decrease the feeding rate. Importantly, ease of mosquito manipulation during manipulations such as dissection with sharps may improve the safety of experiments and this should be taken into account when choosing the design and devising procedures. Other options can include class II or III MSCs – though airflow may prevent efficient feeding and increase escape risks, but this setup is not ideal for dissections given where microscopes or cameras need to be placed.

If HEPA-filtered isolators cannot be used and/or to facilitate experiments and manipulation of arthropods, respirator masks such as filtering facepieces (FFP) 3 masks (Figure 11c) or the use of Purifying Air Powered Respirators (PAPR, Figure 11d) should be assessed. Nevertheless, all steps involving opening mosquito containers should ideally be performed in glove box to limit the escape of infected mosquitoes into the CL3 insectary.

Risk assessments need to be carried out for each setting and work, and procedures (SOPs) were established. Pathogen-associated risks need to be considered individually, with pathogen transmissibility (aerosol transmission) as a relevant factor. Specifically, vector behavior (*e.g*. flying, biting habits, etc.) need to be accounted for. Again, national biosafety rules for handling of pathogens may influence which processes and procedures are to be implemented.

### Pathogen inactivation and disposal of insectary waste

Waste inactivation is generally performed for HG2-infected waste except that solid/liquid waste is autoclaved within the CL3. High volumes of liquid waste can be decontaminated by chemical or thermal effluent treatment via an effluent treatment plant.

Only inactivated samples (*e.g*. vector tissues) can be transported outside the CL3 laboratory for further processing and according to specific and local regulations.

### Movement of samples within CL3 and from CL3 to CL2 laboratories

Infectious samples to be transported within CL3 must be double contained (*e.g*. tubes in transport box). Inactivated samples can be transferred from CL3 to CL2 areas for further analysis. Prior to the movement of material, disinfection and inactivation of samples are required. For this, the outer surfaces of containment/containers must be wiped with disinfectant or dipped in disinfectant according to biosafety regulations. Infectious samples can be inactivated through a variety of procedures that must be previously risk assessed.

### Personal safety measures

PPE including full body suits (head covered), safety spectacles, shoe covers or specific CL3 shoes and two pairs of gloves are required. CL3 entry and exit procedures should clearly state in which order and where (dirty vs clean lobby area) PPE should be put on and removed. For arthropod manipulation, such as dissection, where there is a higher risk of worker exposure to infected material (*e.g*. projections), FFP3 masks or PAPRs should be considered as well as wearing disposable apron and sleeves (on top of a disposable body suit) if not using an HEPA-filtered isolator.

Working in a CL3 environment presents a potentially serious risk to all personnel, therefore, a high level of good microbiological practice and training for pathogen infections of arthropods is required. Activities should be monitored at all times during the presence of staff in the facility, and lone working should be proscribed.

Use of glassware should be kept to a minimum and where possible, plastic alternatives should be used. We do recommend using glass Petri dishes for holding anesthetized mosquitoes on ice as they better conduct cold temperatures and are less prone to accidental movement in the ice bucket. A filter paper disk can be placed at the bottom of the petri dish to avoid condensation and damage of arthropods during their handling. Sharps (forceps, scissors, syringes and capillaries, glass slides and coverslips, and dissection cups) should be enclosed in their dedicated sealable container in the CL3 Insectaries and labeled as sharps. When in use, the sharps are placed so that they will not come into contact with the users’ hands. When manipulating forceps/sharps, hands are never crossed to avoid injuries. Puncture-resistant gloves should be worn when handling sharps, such as the capillary needles used for microinjection systems (such as Nanoject, Drummond Scientific Company, Broomall, PA, US).

As previously, pregnant or breastfeeding personnel need to have specific risk assessments carried out before any work in the CL3 insectaries.

### Disinfection of the CL3 insectaries

For emergency disinfections following a spillage, escape of mosquitoes, routine disinfection to allow servicing (once a year is generally enough), change of microorganisms and commission/decommissioning the laboratory, the CL3 should be fumigated (either with formaldehyde, vaporized hydrogen peroxide [VHP] or chlorine dioxide). Sealability of the CL3 or individual rooms therein is therefore paramount to avoid any leakage of these gases. Importantly, the surfaces need to be thoroughly cleaned after fumigation (usually not required for VHP, although a good practice) to avoid any subsequent negative influence on experimenters and mosquitoes in CL3. It is important that pipes/vents are considered when sealability is assessed.

### Emergency measures

The key concern in the CL3 insectary is the escape of infected arthropods and exposure to pathogens. Individual escapees or low numbers can be dealt with easily by direct killing (handheld zappers or fly catchers are useful); however, this must be followed by decontamination procedures if arthropods were potentially infectious. This can be classed as exposure if escapes take place in the insectary room rather than contained environment such as an isolator. If the escapees cannot be crushed or if larger numbers of insect escapees are found, it may necessitate fumigation of the facility or wait for the arthropods to die (ideally lock room; shifting temperature to higher or lower temperatures may be considered) before reentering the CL3 insectaries. However, survival must be tested in each new environment, and disinfection may be necessary to eliminate risks associated with the pathogen. Clear labeling of rooms or equipment (isolator, climatic chamber, etc.) to establish a perimeter of caution and prevent any risk to other users of the facility is necessary. Spills with pathogen-containing materials such as blood need to be considered, for example, decontamination with inactivating agents or fumigation of the facility depending on volume and circumstances. An emergency plan, explaining actions for different incidents or undesirable events (power failure, autoclave failure, no operation of effluent decontamination system, etc.) should be written in advance (part of COP) and implemented.

In order to facilitate the workflow for accessing CL3 laboratories, a checklist summarizing all the standard procedures required in relation to the topics analyzed in these guidelines is provided (Supplementary File 2, Table S2). This checklist can be shared with the staff working in this type of environment.

## General considerations

### Review and maintenance

Structures and procedures are critical to safe and efficient running of CL2 and CL3 insectaries. Risk assessments and procedures (SOPs), regardless of containment level, should be reviewed at least once a year, and at least when new techniques, arthropods or pathogens are introduced or following incidents and accidents. It is likely that procedures, especially in new insectaries, need to be adapted and tests are recommended before procedures are finalized. Structures encompass the physical envelope of the insectary (walls/windows/doors) but also heating, water, electricity, light and equipment (environmental chambers, blood feeding devices, devices for proper waste treatment, etc.). It is recommended that maintenance contracts are agreed to assess the integrity of structures (universities frequently have specialized staff) and equipment verified as regularly as requested by the manufacturer/warranty conditions to ensure good functioning. In addition, quality certifications or accreditations will impose specific deadlines regarding such verification or calibration operatives.

### Training

Following theoretical training and lectures on specific aspects of biosafety related to the activities to be carried out.

**CL2**: Demonstration of procedures and then at least once under supervision or until competent with procedures. For work with mosquitoes infected with HG2 pathogens, competence to work with non-infected mosquitoes should be demonstrated.

**CL3**: Experience at CL2 level should be a requirement. Training to be provided in specific techniques by defined numbers of sessions, first by shadowing experienced staff and then under supervision. Competence is verified under all circumstances by senior staff.

Refresher training should be offered to staff who have not been working in the facility following initial training (local requirements may vary, but, for example, no active work for 6 months could be used as cut-off). This should be discussed with facility supervisors to define needs. Training records should be kept according to local or national legislation.

### Vaccinations and travel restrictions

Where possible, vaccination should be offered, either for work with the pathogens or before travel to affected areas. Moreover, blood sources (human, etc.) may make vaccination mandatory. If medical treatment is available for a given pathogen, this may also be integrated into a response plan. It is recommended not to work in insectaries with anopheline mosquitoes for 6 weeks if returning from areas where malaria may be present.

For arboviruses, it is recommended not to enter insectaries for a week following return from areas where pathogens, which can be transmitted by vectors in the facility, are endemic or currently being transmitted. If no symptoms are present after this point, the workers may reenter the facility. Self-monitoring is essential, and potentially medical investigation if infection is suspected (for example, after bites, length of stay in affected area, etc.). When working with animal pathogens (*e.g*. Rift Valley fever virus), it may be recommended not to visit facilities with potentially susceptible animals following experiments for a duration that should be decided on a virus-specific risk assessment basis. Such assessments should be carried out for pathogens and insect species to be studied at each location on a case-by-case basis.

## Conclusions

We expect this document to act as a reference point for regulations, procedures and techniques that need to be changed or improved, as well as novel techniques and novel safety-related questions that need to be addressed. As such, we also aim to extend it in the near future with advice on other arthropod vectors that require adaptation of methodologies from the one described in this version. This document and standardized protocols for research with vectors are available at https://infravec2.eu/project-documents/.

## Supplementary Material

Supplemental Material
