# Supplementary material for: Infravec2 guidelines for the design and operation of containment level 2 and 3 insectaries in Europe
Source: Pathog Glob Health. 2022 Aug 22;117(3):293–307. doi: 10.1080/20477724.2022.2108639 (PMC10081053; doi:10.1080/20477724.2022.2108639)
Supplement: Supplemental Material [file YPGH_A_2108639_SM3574.zip › Supplementary file 2.docx]

*Table S1: CL2 Facility Safety Checklist.*

| CL2 FACILITY SAFETY CHECK LIST | Yes | | No | | If no….. | |
| --- | --- | --- | --- | --- | --- | --- |
| **Design** |  | |  | |  | |
| - Access restricted to users (PIN keypad or physical key). |  | |  | |  | |
| - Presence of vector control measures (sticky traps, insect-o-cutor, light traps, etc…) |  | |  | |  | |
| - Access to autoclave in building, and incinerator or tissue digester if required |  | |  | |  | |
| - Validated procedures for inactivation and waste disposal |  | |  | |  | |
| - Record keeping for risk assessments, COSSHs, SOPs, staff training records and other relevant records |  | |  | |  | |
| - Standard operating procedures (SOPs) should, regardless of CL, be reviewed at least once a year |  | |  | |  | |
| - New procedures need to be adapted and tests are recommended before SOPs are finalised |  | |  | |  | |
| - Antechamber or preparation room |  | |  | |  | |
| - Sink for hand washing plus water supply |  | |  | |  | |
| - Rearing room |  | |  | |  | |
| - Working with GM or with HG2 pathogens infected arthropods? Lobby with double doors /air curtain/lobby at +4°C |  | |  | |  | |
| - Safe storage for biological material (e.g. locking freezers) |  | |  | |  | |
| - Fridge/freezer or CO_2_ to anesthetise arthropods |  | |  | |  | |
| - Check for any potential escape routes for arthropods (e.g. nets on ventilations, drainage system) |  | |  | |  | |
| - Barriers (e.g. appropriated mesh) for relevant arthropods (e.g. exotic species) or cooling system (to +4°C) |  | |  | |  | |
| **Design with environmental chambers (for rearing large number of arthropods)_high risk of fungal or/and bacteria contamination** |  | |  | |  | |
| - Alcohol or solvents/cleaning products (avoid misuse to prevent adverse effect on arthropods) |  | |  | |  | |
| - Appropriated benching material, wall coatings or materials and specialist paint (colour white is desirable to spot possible escaped arthropods) to allow decontamination |  | |  | |  | |
| - Appropriated light control (e.g. obscuration of windows; timer for artificial lights) |  | |  | |  | |
| - Appropriated humidity and temperature control (plus probes and remote alarming) |  | |  | |  | |
| - Access to water source (water gun) |  | |  | |  | |
| - Servicing of the humidifying system/chamber on annual base |  | |  | |  | |
| **Design with environmental chambers (requires allocated space)** |  | |  | |  | |
| - Regular maintenance by users |  | |  | |  | |
| - Water level of humidifier system (can be automatised if linked to deionised water tap with at best a flush like system for auto filling) |  | |  | |  | |
| - Removal of waste water (waste water draining system can be designed in the room floor if new room designed) |  | |  | |  | |
| - See through chamber |  | |  | |  | |
| **Facility access** |  | |  | |  | |
| - Is the staff trained for working under CL2 containment? (mock training with noninfected arthropods before working with infected ones) |  | |  | |  | |
| - Is vaccination for pathogen in use available? |  | |  | |  | |
| **Personal safety measures** | |  | |  | |  |
| - Lab coat, gloves | |  | |  | |  |
| - Sharps (forceps, scissors, syringes and capillaries, glass slides and coverslips, dissection cups) should be enclosed in their dedicated sealable container and labelled as “sharp” | |  | |  | |  |
| - Goggles or masks depending on local risk assessments for the procedure and pathogen | |  | |  | |  |
| **Breeding pathogen-free arthropods** | |  | |  | |  |
| - Rearing pans | |  | |  | |  |
| - Protection for the escape of emerged adult (e.g. nets on top of rearing pans, plastic transparent cover) | |  | |  | |  |
| - Mesh cages (check mesh size is appropriated to the arthropod species) | |  | |  | |  |
| - Labelling of pans and cages | |  | |  | |  |
| - Check the source of blood for routine feeding (which organism, specify RA and SOP) | |  | |  | |  |
| **Performing arthropod infection with HG2 pathogens (MUST be separated from the pathogen-free insectary)*** | |  | |  | |  |
| - Are RA/COSHH/SOPs for your specific work available? | |  | |  | |  |
| - Do you have all the required containment (1-3 layers)? | |  | |  | |  |
| - Have you counted all the arthropods before infection and after manipulation? | |  | |  | |  |
| - Have you labelled all the arthropods containers? | |  | |  | |  |
| - Do you have the appropriate disinfectant (e.g. Virkon) at the right concentration? | |  | |  | |  |
| - Do you have an electrical aspirator for arthropods? This should be avoided to aspire flying arthropods when infected but chilled or CO_2_ anesthetised | |  | |  | |  |
| - Chill table (or fridge and ice buckets) or CO_2_ flypad | |  | |  | |  |
| - Knowledge on the pathogen-specific risk information available (e.g. pathogen titre, potential for its transmission, route of infection)- linked to RA | |  | |  | |  |
| - Relevant biosafety documentation for pathogen handling | |  | |  | |  |
| - MSC HEPA filtered for pathogen handling | |  | |  | |  |
| - Glovebox or “glove bag” type device for arthropods handling (according to pathogens or experiment) | |  | |  | |  |
| - Respiratory mask and safety glasses (if necessary), disposable aprons and sleeves in case of projection/insect fall during dissection at bench | |  | |  | |  |
| - Appropriated feeding device (e.g. hemotek, feeding chamber into waterbath) | |  | |  | |  |
| - Transport box (if movement between rooms is required) | |  | |  | |  |
| **Insectary waste** | |  | |  | |  |
| - Check with your national and local/or local biosafety regulations. | |  | |  | |  |
| - Liquid waste from infected material: decontamination by autoclaving, chemical or thermal effluent treatment (check for each pathogen what can be safely used to disinfect) | |  | |  | |  |
| - Liquid waste from larval rearing: filtration, o/n at -20°C (or boiled), virkon tablets overnight, discard in normal wastewater system | |  | |  | |  |
| - Solid waste: allocated into autoclave bags, stored o/n at -20°C, autoclaved and/or incinerated (if necessary inactivation process with appropriated disinfectants before storage in autoclave bags**) | |  | |  | |  |
| - Solid waste (animal products): mark the autoclave bags as ABP waste | |  | |  | |  |
| **Disinfection of CL2 insectaries** | |  | |  | |  |
| - Clean surfaces thoroughly after fumigation | |  | |  | |  |
| - Spillage accident: need to be considered and dealt with according to local/national safety practices | |  | |  | |  |
| **In case of arthropod escape (procedure should be written in RA and signs available for staff to let know escapes)** | |  | |  | |  |
| - Direct killing (handheld zappers, or fly catchers are useful) | |  | |  | |  |
| - Fumigation of the facility or test which amount of time arthropods survive without any access to water, for instance | |  | |  | |  |
| - Shifting temperature to higher or lower temperatures | |  | |  | |  |
| **Movement of material from CL2 to CL1** | |  | |  | |  |
| - Have you disinfect the outer surfaces of bags or containers (tubes, transport boxes) to be moved? | |  | |  | |  |
| - Have you inactivated infectious material (by boiling, lysing buffer, fixation, etc.) | |  | |  | |  |
| - Transport container | |  | |  | |  |
| **Have you worked through the check list?** | |  | |  | |  |

*Procedures with infectious agent depend on pathogen and need to be adapted to safety requirements for working with specific agents.

**Disposure of animal by product waste (ABP) has to be checked with local regulations.

*Table S2: CL3 Facility Safety Checklist.*

| CL3 FACILITY SAFETY CHECKLIST | Yes | No | If no….. |
| --- | --- | --- | --- |
| **Design** |  |  |  |
| - Clean and dirty designated and well defined areas |  |  |  |
| - Monitor for negative pressure |  |  |  |
| - Sealed laboratory (secondary containment or barrier) to be regularly checked for leakages |  |  |  |
| - Presence of vector control measures (sticky traps, insectocutor, etc.) |  |  |  |
| - Access to autoclave within the CL3 suite or double-ended |  |  |  |
| - Intruder alarm |  |  |  |
| - Record keeping |  |  |  |
| - Safe storage for biohazards |  |  |  |
| - Transport measures |  |  |  |
| - Any potential escape route for arthropods? |  |  |  |
| - Mesh for drainage/air |  |  |  |
| - Blocking drains |  |  |  |
| - Effluent treatment system |  |  |  |
| - (Air) curtains |  |  |  |
| - Are there any vision panels and/or web cams/CCTV? |  |  |  |
| - See through climatic chamber with humidity and temperature display |  |  |  |
| - MSC class II HEPA-filtered for pathogen handling |  |  |  |
| - Mask or respirator system, alternatively glovebox with HEPA type filter system depending on nature of pathogen or experiment |  |  |  |
| - Glovebox or “glove bag” type device for arthropod handling (according to pathogens or experiment) |  |  |  |
| - Pass-through (such as liquid disinfectant-containing) or airlock systems |  |  |  |
| - Standard operating procedures (SOPs) should, regardless of CL, be reviewed at least once a year |  |  |  |
| - New procedures need to be adapted and tests are recommended before SOPs are finalised |  |  |  |
| **Facility access** |  |  |  |
| - Is the staff trained for working under CL3 containment? Visitors with trained buddy? |  |  |  |
| - User access recognitions available? |  |  |  |
| - “Dead man” available? |  |  |  |
| - Is there a logbook for users? |  |  |  |
| - Is vaccination for pathogen in use available? |  |  |  |
| **Personal safety measures** |  |  |  |
| - Full body suits (head covered), safety spectacles, overshoes and two pairs of gloves |  |  |  |
| - Sharps (forceps, scissors, syringes and capillaries, glass slides and coverslips, dissection cups) should be enclosed in their dedicated sealable container and labelled as “sharp” |  |  |  |
| **Performing arthropod infection with pathogens** |  |  |  |
| - Are SOPs for your specific work available? |  |  |  |
| - Do you have all the required containment (1-3 layers)? |  |  |  |
| - Have you counted all the arthropods before infection and after manipulation? |  |  |  |
| - Have you labelled all the arthropods containers? |  |  |  |
| - Do you have the appropriate disinfectant (e.g. Virkon) at the right concentration? |  |  |  |
| - Do you have an electrical aspirator for arthropods? |  |  |  |
| **Insectary waste** |  |  |  |
| - Check with your national and local/or local biosafety regulations. |  |  |  |
| - Liquid waste: decontamination by autoclaving, chemical or thermal effluent treatment |  |  |  |
| - Solid waste: immersion in inactivating agent (such as Virkon), autoclaved and/or incinerated |  |  |  |
| **Disinfection of CL3 insectaries** |  |  |  |
| - Fumigation (either with formaldehyde, vaporised hydrogen peroxide [VHP] or chlorine dioxide) |  |  |  |
| - Clean surfaces thoroughly after fumigation |  |  |  |
| - Spillage accident: need to be considered and dealt with according to local/national safety practices |  |  |  |
| **In case of arthropod escape** |  |  |  |
| - Direct killing (handheld zappers, or fly catchers are useful) |  |  |  |
| - Fumigation of the facility |  |  |  |
| - Shifting temperature to higher or lower temperatures |  |  |  |
| **Movement of material from CL3 to CL2** |  |  |  |
| - Have you disinfect the outer surfaces of bags or containers to be moved? |  |  |  |
| - Have you inactivated infectious material (by boiling, lysing bugger, fixation, etc..) |  |  |  |
| - No papers or tapes are allowed to be moved from CL3 without previous autoclaving |  |  |  |
| - Transport container |  |  |  |
| **Have you worked through the check list?** |  |  |  |
